# Imaging the “Hot Phase” of a Familiar Left-Dominant Arrhythmogenic Cardiomyopathy

**DOI:** 10.3390/genes12121933

**Published:** 2021-11-30

**Authors:** Marta Rubino, Alessandra Scatteia, Giulia Frisso, Giuseppe Pacileo, Martina Caiazza, Carmine Emanuele Pascale, Pasquale Guarini, Giuseppe Limongelli, Santo Dellegrottaglie

**Affiliations:** 1 Cardiovascular MRI Laboratory, Division of Cardiology, Ospedale Medico-Chirurgico Accreditato Villa dei Fiori, 80011 Acerra, Naples, Italy; rubinomarta@libero.it (M.R.); a.scatteia@gmail.com (A.S.); carmineemanuele.pascale@gmail.com (C.E.P.); guarini@iol.it (P.G.); sandel74@hotmail.com (S.D.); 2 Inherited and Rare Disease Unit, Department of Translational Medical Sciences, Università degli Studi della Campania “Luigi Vanvitelli”, 81100 Caserta, Italy; gpacileo58@gmail.com (G.P.); martina.caiazza@yahoo.it (M.C.); 3 Department of Molecular Medicine and Medical Biotechnologies, University Federico II, 80138 Napoli, Italy; gfrisso@unina.it; 4 CEINGE, Advanced Biotechnologies, 80145 Napoli, Italy; 5 Zena and Michael A. Wiener Cardiovascular Institute/Marie-Josee and Henry R. Kravis Center for Cardi-ovascular Health, Icahn School of Medicine at Mount Sinai, New York, NY 10029, USA

**Keywords:** myocarditis, arrhythmias, cardiac magnetic resonance imaging

## Abstract

We describe the case of a young man with an initial diagnosis of acute myocarditis that was finally recognized as a familial left-dominant arrhythmogenic cardiomyopathy. The diagnostic process was also based on demonstration, serial cardiac magnetic resonance imaging, and typical patterns of myocardial damage, including features of the disease’s inflammatory “hot phase”.

## 1. Introduction

Arrhythmogenic cardiomyopathy is currently defined as a (genetic or non-genetic) heart muscle disorder characterized by progressive ventricular myocardial damage with fibrofatty replacement, which may represent the substrate for malignant ventricular arrhythmias [1]. Although in the past it was believed to be a disease involving only the right ventricle (RV), a broader spectrum of phenotypic expression has subsequently been identified, including biventricular and left-dominant variants [2]. In many cases, diagnostic recognition may be difficult and is reached using a continuously updated scoring system where a series of genetic, morpho-functional, electrocardiographic, arrhythmic, and tissue disease characteristics are considered [1,3]. Clinical presentation is generally dominated by arrhythmic events, or less commonly by symptoms related to ventricular dysfunction. In rare (or more likely in under-recognized) cases, however, affected patients experience one or more episodes of acute chest pain, with evidence of myocardial damage as presenting features (the so-called “hot phase”) [2].

## 2. Case Report

**Figure 1:**
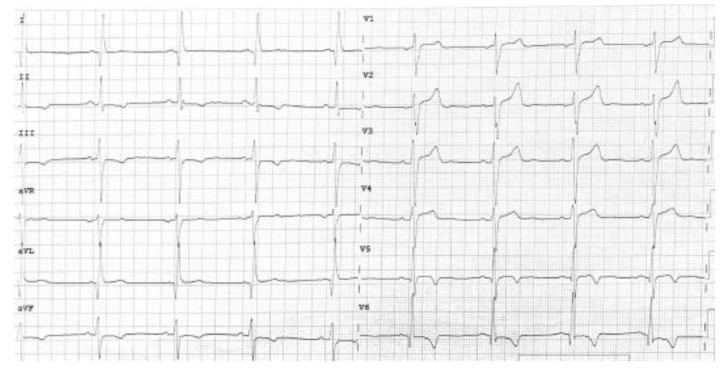
12-leads ECG showing T wave inversion in the inferolateral leads.

**Figure 2:**
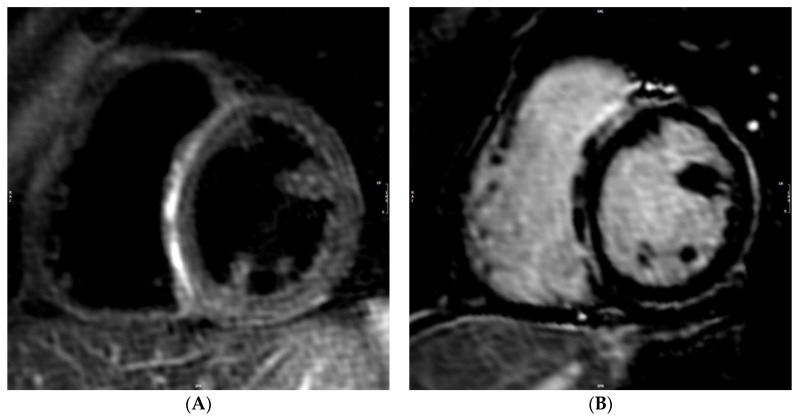
(**A**) STIR T2 weighted short-axis mid-ventricular image showing an area of high signal intensity in the interventricular septum (arrows). (**B**) LGE short-axis mid-ventricular image showing mid-wall enhancement in the interventricular septum (arrowheads).

**Figure 3:**
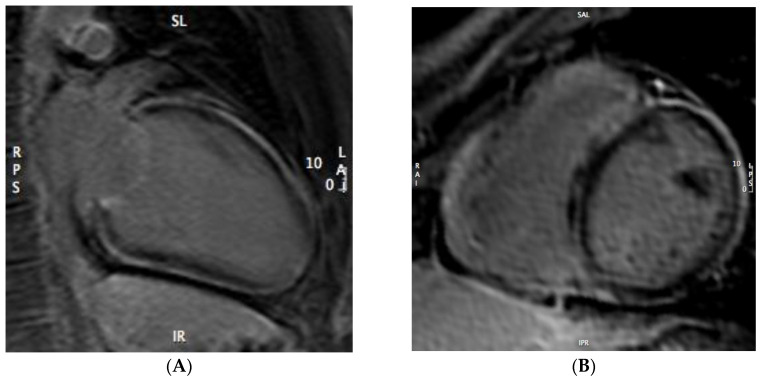
2-chamber (**A**) and mid-ventricular short-axis (**B**) LGE images showing subepicardial circumferential enhancement involving the entire left ventricle (arrows).

A 23-year-old man was admitted to our cardiomyopathy clinic for repetitive ventricular ectopic beats. He was hemodynamically stable with no other relevant symptoms. He never experienced syncope and was unaware of any case of cardiomyopathy or sudden cardiac death in his family. Remarkably, his medical history included an episode of acute myocarditis one year before. At that time, he was admitted to the emergency department of a different hospital with chest pain, troponin rise, and T wave inversion in the inferolateral leads on ECG (Figure 1). An urgent coronary angiogram revealed normal coronary arteries. Then, a cardiac magnetic resonance (CMR) was performed, showing a non-dilated left ventricle (LV) with low-normal ejection fraction (EF), as well as normal RV dimensions and function. T2-weighted images highlighted the presence of mid-wall myocardial edema involving the interventricular septum, where mid-wall late gadolinium enhancement (LGE) was also noted on post-contrast images (Figure 2). Endomyocardial biopsy was proposed, though the patient did not provide informed consent. The patient was discharged with a diagnosis of acute myocarditis, with a recommendation for close clinical follow-up. When re-assessing the patients at his 1-year follow-up, echocardiography showed an initial reduction of LV EF, with an area of hypo-akinesia involving the lateral wall, and preserved RV dimensions and function. A new CMR study was performed, which confirmed the mildly reduced LV EF with no evidence of myocardial edema. Post-contrast images, however, revealed a diffuse circumferential subepicardial LGE involvement of the LV myocardium (Figure 3).

**Figure 4:**
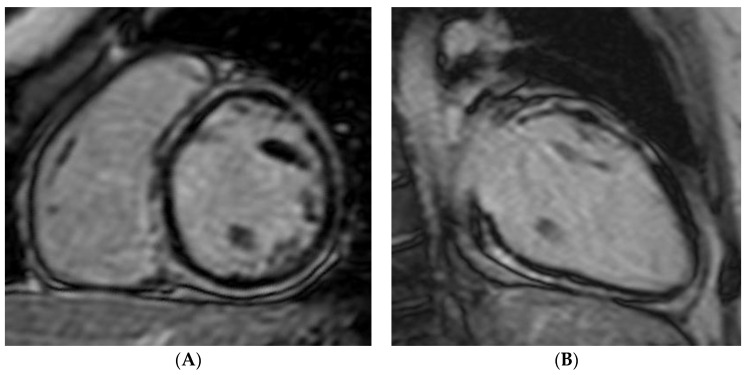

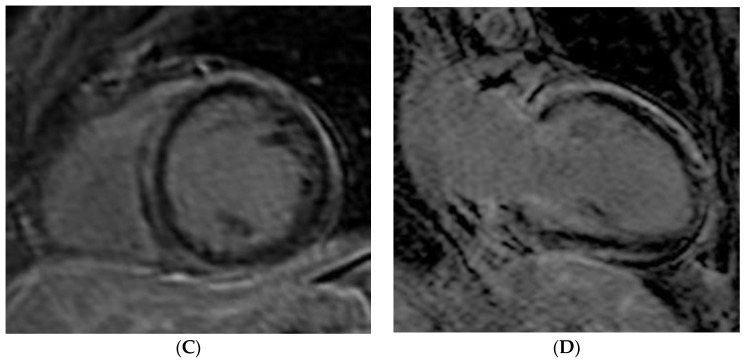
LGE 2 chamber (**A**) and short-axis (**B**) images of the patient’s sister and mother (**C**,**D**) showing subepicardial circumferential late enhancement similar to that found in the proband.

To exclude a left-dominant variant of arrhythmogenic cardiomyopathy, in which this LGE pattern has been reported with CMR, genetic testing and accurate family screening were then performed. His 56-year-old mother and 30-year-old sister, both asymptomatic, were also found to have inverted T waves in the inferolateral leads on ECG and a mildly reduced LV EF on echocardiogram. Performing CMR on those two subjects, a pattern of LGE very similar to the one detected in the proband was detected (Figure 4). No relevant clinical findings were identified by exploring the paternal side of the family. To our knowledge, this was the only documented case of a “myocarditis-like” onset of arrhythmogenic cardiomyopathy among the family members.

Molecular testing was carried out by analyzing a panel of target genes through an NGS-based procedure. The MAF threshold was set to 5% using Illumina Variant Interpreter Software. Genetic testing identified a heterozygous variant in DSP (c.5428C>T, p.Gln1810Ter).

According to the American College of Medical Genetics (ACMG), the variant was classified as likely pathogenic (class IV).

**Figure 5:**
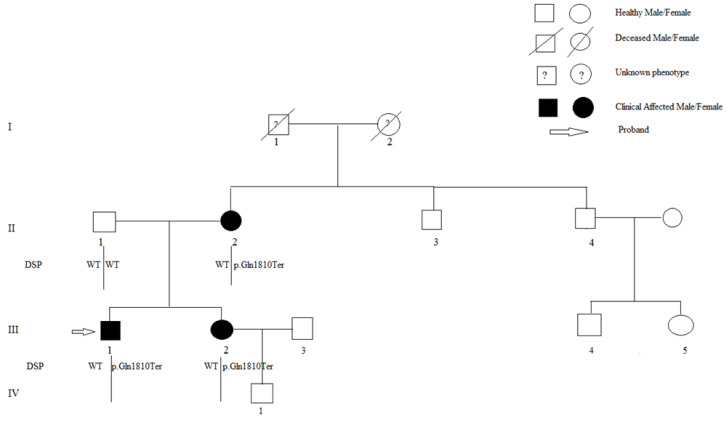
Pedigree of the family of propositus. Genotype and phenotype are defined according to the legend inset. Open symbols represent subjects with a negative genotype and phenotype. II-2, III-1, III-2: Arrhythmogenic cardiomyopathy; I-1, I-2: Unknown phenotype.

The same mutation was found in the patient’s relatives with a positive phenotype (Figure 5), and a diagnosis of familiar left-dominant arrhythmogenic cardiomyopathy was finally made. The patient, as well as his mother and sister, started therapy with β-blocker drugs, and the proband also received an implantable cardioverter-defibrillator.

## 3. Discussion

Desmosomes are specialized adhesion junctions. They provide mechanical connections between cardiomyocytes and mutations in gene encoding proteins involved in the desmosome apparatus (such as plakophilin-2 [PKP-2], desmoplakin [DSP], desmoglein–2 [DSG–2], etc.), and have been identified as responsible for arrhythmogenic cardiomyopathy in about 50% of cases [4]. In affected subjects, dysfunction of intercellular adhesion junctions is responsible for developing the disease phenotype, including loss of efficient electrical coupling (with consequent increased arrhythmic burden), fibrofatty replacement of the myocardium, and progression to ventricular dysfunction [5].

In cases of arrhythmogenic cardiomyopathy associated with DSP mutation, as compared to other genetic patterns, the phenotypic expression tends to include a more common and severe LV involvement [6,7]. In these patients, histology and CMR frequently reveal extensive myocardial fibro-fatty replacement, typically occurring as a circumferential band involving the outer LV myocardial layer and ultimately resulting in LV dysfunction [8].

During the last decades, a possible role for inflammation in the development and progression of myocardial damage in patients with DSP mutations has been postulated, supporting the hypothesis that inflammatory dysregulation could contribute to phenotypic disease progression [9]. In terms of pathophysiology, possible variable links between myocarditis and arrhythmogenic cardiomyopathy have been proposed, and clusters of familial-recurrent myocarditis associated with DSP mutations have been increasingly described [10,11]. However, whether myocarditis is a primary cause of the development of the cardiomyopathic phenotype or the result of cellular and humoral reaction to cardiomyocytes dysfunction and death is not clearly understood. Some cardiotropic viruses (i.e., enterovirus and adenovirus) have been identified in the myocardium of patients with arrhythmogenic cardiomyopathy and are considered to play a potential pathogenetic role in the disease, as the activation of inflammatory mediators could damage the cardiac adherent junctions and the T-tubule system [12]. In addition, it has been suggested that genetic variation in structural proteins may increase the susceptibility of the myocardium to infections [13].

Recent observations further support the existence of a peculiar form of arrhythmogenic cardiomyopathy in patients with DSP mutations, presenting a clinical course that frequently includes episodic myocardial injuries, anticipating the occurrence of LV myocardial fibrosis, and dysfunction [14,15]. Detectable myocardial damage would result from a sub-continuous process of cellular death and replacement, with possible clinical episodes of acute damage, also known as *hot phases* of the disease that in many cases resemble episodes of acute coronary syndrome or myocarditis.

The use of steroid treatment is well supported in the context of fulminant myocarditis related to a specific disease (i.e., giant cell myocarditis), while the benefit of immunomodulatory therapies has not shown any benefit in long term outcome studies in patients who have not performed endomyocardial biopsy [16].

A recent study [17] demonstrated the presence of anti-heart autoantibodies in patients with arrhythmogenic cardiomyopathy, both in the majority of familial and in almost half of sporadic cases. Furthermore, the presence of an autoimmune pattern was associated with disease severity, although longitudinal studies are needed to assess the impact of autoimmunity on the prognosis of these patients.

Despite compelling evidence indicating the role of inflammation in ACM, immunomodulatory therapies are seldom used. In a small case series of pediatric AC patients, a decrease in hospital stay was demonstrated, although steroid therapy seemed not to prevent disease progression or affect outcomes [18]. There are insufficient data regarding using more selective monoclonal antibodies, such as canakinumab, which has been demonstrated to reduce CV events in the CANTOS trial [19]. However, the role of monoclonal antibodies could represent an important therapeutic target for future investigations, as demonstrated by preclinical studies [20].

## 4. Conclusions

The present report highlights the possibility that clinically suspected acute myocarditis may represent, instead, the *hot phase* of genetically-determined arrhythmogenic cardiomyopathy. High accuracy and reproducibility in the assessment of ventricular morphology and function, together with the ability to provide tissue characterization, make CMR the ideal technique in assisting the diagnosis of both arrhythmogenic cardiomyopathy and myocarditis. To correctly guide further management, the possibility of an association between these two conditions should be kept in mind when performing serial CMR scans after a clinically evident episode of acute non-ischemic myocardial damage.

## Data Availability

The data that support the findings of this study are available from the corresponding author upon reasonable request.
